# Successful Pregnancy After Aspiration and Re-transfer of a Prematurely Released Embryo During a Frozen Embryo Transfer: A Case Report

**DOI:** 10.7759/cureus.107374

**Published:** 2026-04-20

**Authors:** Limia Ibrahim, Michael Fakih

**Affiliations:** 1 Obstetrics and Gynecology/Reproductive Medicine, Bourn Hall Fertility Clinic, Abu Dhabi, ARE; 2 Obstetrics and Gynecology/Reproductive Medicine, Fakih IVF Centre, Abu Dhabi, ARE; 3 Obstetrics and Gynecology/Reproductive Medicine, School of Medicine, American University of Beirut, Beirut, LBN; 4 Obstetrics and Gynecology/Reproductive Medicine, Yale University, New Haven, USA

**Keywords:** aspiration and re-transfer, assisted reproductive technologies, embryo bubble, embryo location, embryo transfer, in vitro fertilization (ivf), premature embryo release, successful pregnancy, transfer technique, ultrasound-guided embryo transfer

## Abstract

Embryo transfer (ET) is a critical step in in vitro fertilization (IVF) and plays a pivotal role in determining implantation and pregnancy outcomes. Despite the routine use of soft catheters and real-time ultrasound guidance to ensure accurate embryo placement, procedural complications may occasionally occur. Premature release of an embryo into the lower uterine cavity or the utero-cervical junction is a rare event that may compromise implantation success.

We report the case of a 36-year-old woman undergoing frozen embryo transfer (FET) of a day 5 blastocyst. Immediate post-transfer ultrasound visualization demonstrated displacement of the embryo bubble into the utero-cervical junction. Prompt aspiration of the prematurely released blastocyst was performed, followed by reloading and re-transfer into the optimal fundal region under ultrasound guidance.

The patient achieved a positive human chorionic gonadotropin (β-hCG) result and developed an intrauterine pregnancy that progressed to term. An unexplained intrauterine fetal death occurred at 39 weeks’ gestation.

This case emphasizes the importance of continuous ultrasound monitoring during ET, rapid recognition of embryo misplacement, and meticulous coordination between clinical and embryology teams. Successful implantation and continuation of pregnancy may still be achieved following immediate retrieval and re-transfer of a prematurely released blastocyst.

## Introduction

In vitro fertilization (IVF) treatment involves multiple processes to achieve a successful outcome [1]. Embryo transfer (ET) is a critical step in IVF and plays a pivotal role in determining implantation and pregnancy outcomes [2]. Despite the routine use of real-time ultrasound guidance to ensure accurate embryo placement and optimal technique, procedural complications may occasionally occur [3,4]. Premature release of an embryo into the lower uterine cavity or the utero-cervical junction is an event that may compromise implantation success [5].

We report a case of a 33-year-old woman undergoing frozen embryo transfer (FET) of a day 5 blastocyst. During ET under real-time ultrasound guidance, the embryo bubble was visualized migrating into the utero-cervical junction rather than the intended fundal uterine cavity [6,7]. Prompt aspiration of the prematurely released blastocyst was performed, followed by reloading and re-transfer into the optimal fundal region under ultrasound guidance [5,7].

The patient achieved a positive pregnancy result. The conception progressed to an intrauterine pregnancy that reached term. During antenatal follow-up with the obstetrician, an unexplained intrauterine fetal death was diagnosed at 39 weeks of gestation.

This case emphasizes the importance of continuous ultrasound monitoring during ET, rapid recognition of embryo misplacement, proper embryo handling in managing procedural complications during ET, and meticulous coordination between clinical and embryology teams. Successful implantation and continuation of pregnancy may still be achieved following immediate retrieval and re-transfer of a prematurely released blastocyst [4,5].

## Case presentation

Couple history

A couple presented with secondary infertility of two years’ duration. The female partner was a 33-year-old woman, accompanied by her 36-year-old husband during evaluation. The patient had been pregnant three times and had delivered three children vaginally. Her last childbirth was two years ago. Upon investigation, infertility was found to be due to factors from both partners. On the female side, testing revealed a low ovarian reserve, indicated by an anti-Müllerian hormone (AMH) level of 0.854 ng/mL [8]. The male factor involved astheno-teratozoospermia, as detailed in Table 1 [9].

**Table 1:** Male semen analysis parameters (astheno-teratozoospermia)

Parameter	Value	Normal Range
Sperm Concentration	45 million/mL	≥ 16 million/mL
Total Sperm Count	90 million	≥ 39 million per ejaculate
Progressive Motility	24%	≥ 30%
Normal Morphology	3%	≥ 4%

The couple underwent an IVF treatment cycle in March 2021, which resulted in the development of three embryos.

**Figure 1:**
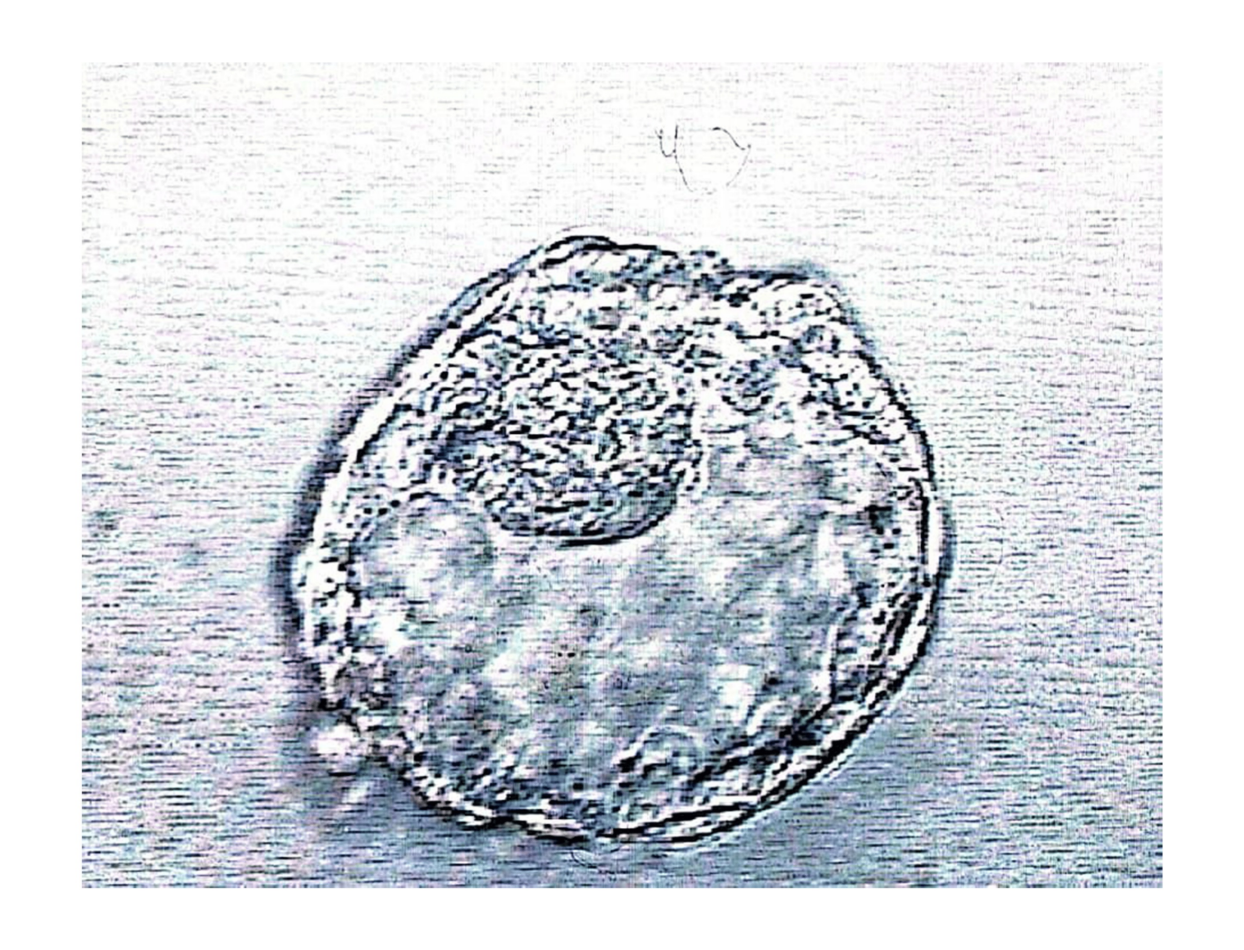
Day 5 frozen pretransfer embryo, grade 5BB

A day 5, grade 5BB blastocyst was cryopreserved following a trophectoderm biopsy and identified as euploid following preimplantation genetic testing for aneuploidy (PGT-A) (Figure 1).

The FET cycle started on July 15, 2022 (day 2 of the menstrual cycle), with endometrial priming using estradiol followed by progesterone. Hormonal assay results performed one day before the transfer are shown in Table 2.

**Table 2:** Pre-FET hormonal profile FET: frozen embryo transfer

Hormone	Value	Normal Range (Approx.)
Estradiol (E2)	218 pg/mL	150-250 pg/mL (luteal phase)
Follicle-Stimulating Hormone (FSH)	3.7 IU/L	1.5-10 IU/L
Luteinizing Hormone (LH)	8.15 IU/L	1.5-8 IU/L (mid-luteal phase)
Progesterone (P4)	7.3 ng/mL	5-20 ng/mL (luteal phase)

History

On July 28, 2022, an FET was scheduled. Under ultrasound guidance, the ET procedure began with the insertion of the outer sheath of the transfer catheter through the cervical canal into the mid-uterine cavity. During the passage of the embryo-loaded inner catheter, a spontaneous release of the hyperechogenic “embryo bubble” was noted before reaching the desired location within the uterine cavity. The embryo bubble quickly descended and settled just above the cervico-uterine junction. The catheter assembly was promptly withdrawn and inspected by the embryologist, who confirmed that it no longer contained the embryo. A new catheter system was prepared instantly, and the inner catheter was carefully advanced to the site of the hyperechogenic spot. Using gentle negative pressure with a 1 mL syringe, the embryo bubble was successfully aspirated back into the catheter. The catheter was then handed to the embryologist, who verified the embryo’s retrieval. Following a rinse in fresh culture medium and a brief incubation, the embryo was reassessed for structural integrity and viability. Once confirmed, it was reloaded into the catheter and successfully transferred to the mid-uterine cavity under ultrasound guidance.

Main Results and the Role of Chance

**Figure 2:**
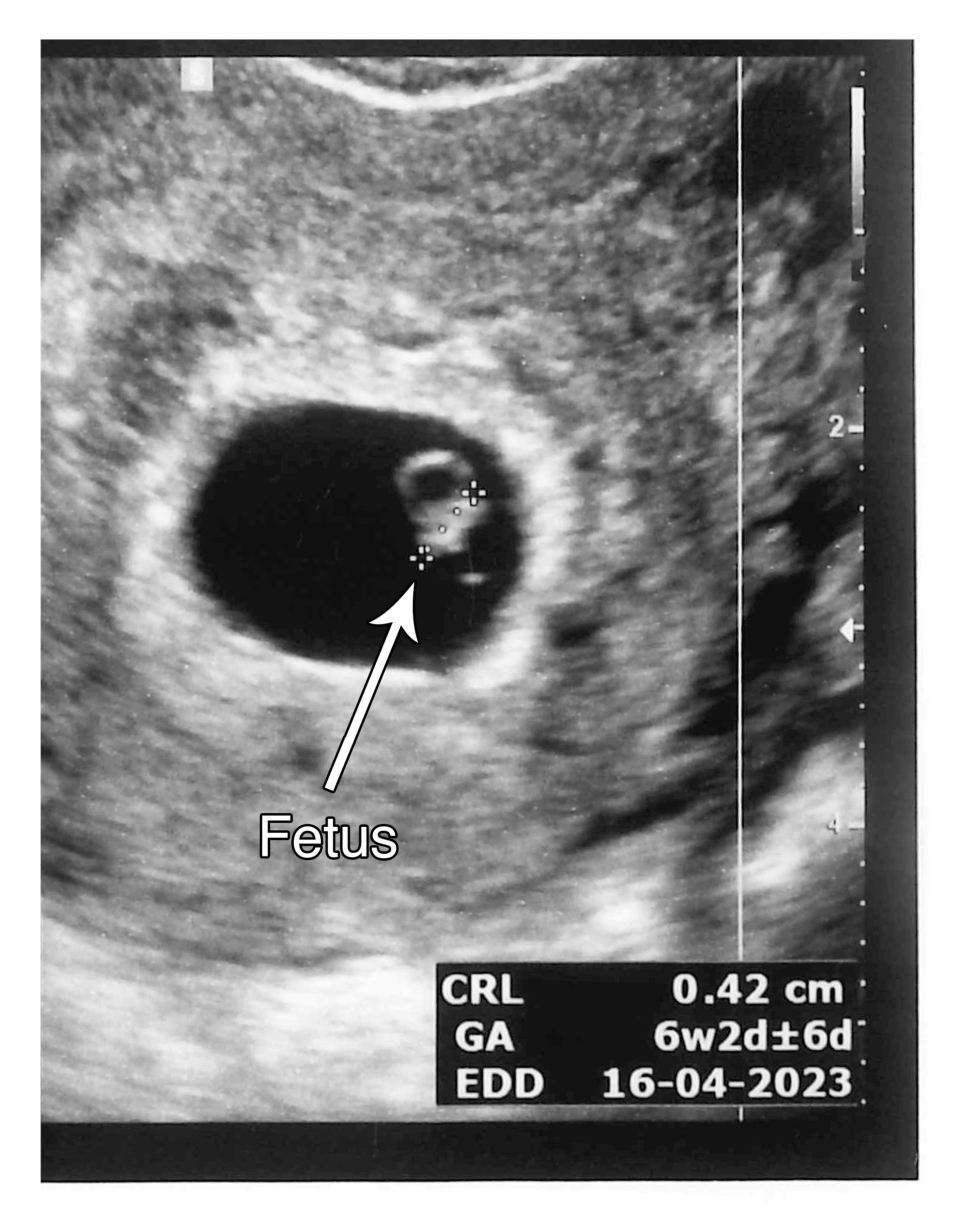
TVUS image of fetus (CRL 0.42 cm = 6 weeks and 2 days) TVUS: transvaginal ultrasound; CRL: crown-rump length

A pregnancy test conducted 10 days post-transfer (on August 7, 2022) confirmed a positive result, with a serum β-hCG (human chorionic gonadotropin) level of 103 mIU/mL. Serum β-hCG levels following the transfer are shown in Figure 2.

**Figure 3:**
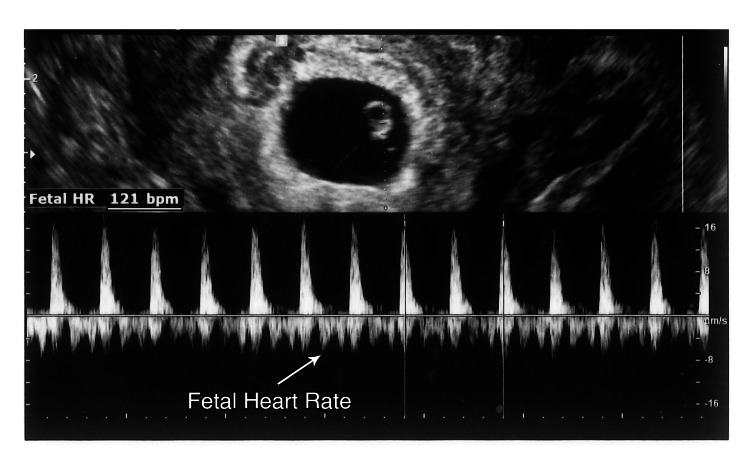
Fetal cardiac activity at 6 weeks 2 days (HR 121 BPM)

**Figure 4:**
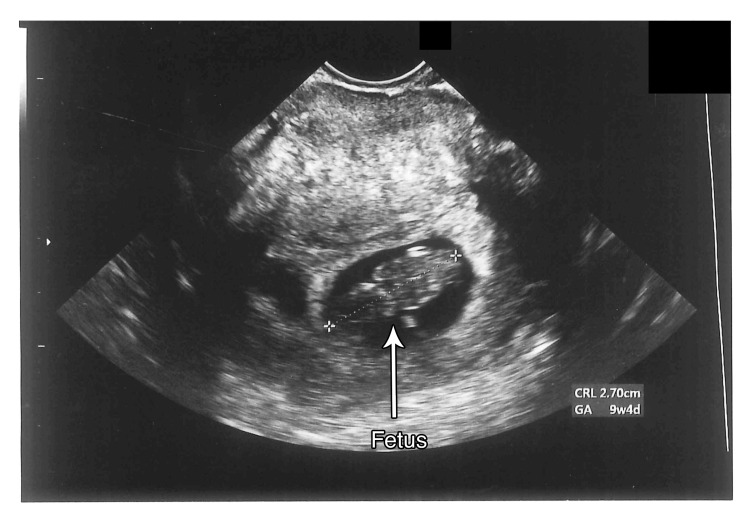
TVUS image of fetus (CRL 2.74 cm = 9 weeks and 4 days) TVUS: transvaginal ultrasound; CRL: crown-rump length

On August 23, 2022 (gestational age (GA): 6 weeks 2 days), a transvaginal ultrasound (TVUS) confirmed a single, viable intrauterine pregnancy, with a crown-rump length (CRL) corresponding to 6w + 2d and a heart rate (HR) of 121 beats per minute (BPM) (Figures 3-4).

**Figure 5:**
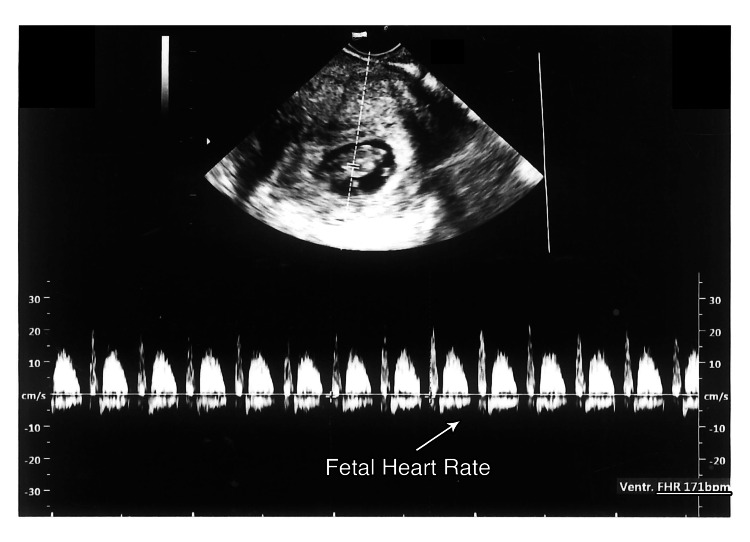
Fetal cardiac activity at 9 weeks 4 days (HR 177 BPM)

A follow-up TVUS, performed on September 15, 2022, at a GA of 9 weeks 4 days, confirmed continued viability, showing a CRL of 2.74 cm, consistent with 9 weeks 3 days, and a fetal HR of 177 BPM (Figure 5).

At 10 weeks, the patient was referred to an obstetrician for antenatal care. Her antenatal follow-up was uneventful until term. However, during a routine antenatal visit at 39 weeks, an intrauterine fetal death was diagnosed. Subsequent investigations revealed the fetal demise to be unexplained.

## Discussion

Accurate placement of the embryo within the upper to middle portion of the uterine cavity - approximately 10-20 mm below the fundal endometrium - is critical for achieving optimal implantation and pregnancy outcomes [2]. The fundal endometrium is widely recognized as the most favorable site for embryo implantation in IVF, owing to several anatomical, molecular, and physiological advantages.

From a molecular standpoint, the fundal region exhibits strong expression of essential adhesion molecules, such as integrins, which play a key role in facilitating firm embryo attachment [5]. Additionally, the production of growth factors and cytokines - including vascular endothelial growth factor (VEGF) and leukemia inhibitory factor (LIF) - enhances endometrial receptivity by supporting angiogenesis and establishing an optimal blood supply [10]. Hemodynamically, the fundal region demonstrates superior vascular perfusion compared to other uterine areas, providing enhanced oxygenation and nutrient delivery for implantation [5,10].

Hormonally, the fundal endometrium contains a higher density of progesterone receptors, promoting an optimal hormonal environment for embryo implantation [11]. Furthermore, the region is characterized by reduced endometrial wave activity, which contributes to mechanical stability and supports early embryonic attachment [12]. Collectively, these factors make the fundal endometrium the preferred target site for embryo deposition during IVF procedures.

This case underscores the effective management of premature embryo release, a rare but recognized complication during ET. Such premature expulsion may result from technical factors - such as incorrect catheter loading, suboptimal insertion technique, or excessive and abrupt movements that disrupt embryo positioning [3]. Additional causes include excessive injection pressure, uterine contractions [12], or misalignment of the catheter, which can cause premature dispersion of air bubbles carrying the embryo away from the intended implantation site [12]. Air bubble migration was documented in 12.4% of ET cycles [7]. Endometrial or uterine factors, including excess intrauterine fluid, mucus accumulation, or suboptimal receptivity, may further hinder smooth ET [13]. Prompt identification and correction of these issues are vital to prevent embryo loss and avoid uterine trauma.

In this case, several factors contributed to the successful outcome. Continuous ultrasound guidance enabled real-time visualization of the embryo’s position, allowing rapid retrieval and precise retransfer. The hyperechogenic appearance of the embryo on ultrasound facilitated immediate localization, reinforcing the importance of image-guided techniques in ET [5,6]. Close collaboration between the physician and embryologist ensured the retrieved embryo’s viability before retransfer. The use of gentle aspiration minimized mechanical stress, preserving both embryo integrity and endometrial receptivity. Literature consistently supports that meticulous ET techniques, performed under ultrasound guidance, significantly improve implantation and pregnancy outcomes while reducing procedural complications [6,14,15].

This case highlights how careful ultrasound-guided retrieval and retransfer into the optimal uterine segment can rescue embryos and improve implantation outcomes. Moreover, it helps avoid the loss of potentially live embryos and spares patients the financial, physical, and psychological burden of undergoing another IVF cycle. This case reinforces the importance of conducting further studies to evaluate the effect of brief embryo exposure to the uterine cavity during such interventions, which may affect implantation potential or overall pregnancy rates.

## Conclusions

Embryo misplacement during transfer is a known occurrence that can reduce implantation success and increase expulsion risk, leading to embryo loss and eliminating pregnancy. In this case, a misplaced embryo was promptly identified and successfully managed through ultrasound-guided aspiration and re-transfer, which preserved its viability and led to a full-term pregnancy. The use of real-time ultrasound visualization was pivotal in locating the embryo and ensuring precise handling, emphasizing the importance of meticulous technique in assisted reproductive technology (ART). This case highlights the feasibility of embryo aspiration and how careful ultrasound-guided retrieval and re-transfer into the optimal uterine segment can rescue embryos and improve implantation outcomes. Furthermore, it suggests the need for future studies to assess whether brief embryo exposure to the uterine cavity during such interventions affects implantation potential or overall pregnancy rates.
